# Military Hygiene

**Published:** 1889-11-23

**Authors:** 


### SANITATION.

### MILITARY HYGIENE.

Surgeon-General C. A. Gordon, C.B., has published some very interesting notes on medico-military hygiene, ancient and modern, in the *Medical Press*, November, 1889. The author shows what has been done by medical officers from the earliest to the present time, and he observes, "scarcely a compaign has failed, rarely has a great military disaster occurred which could not be attributed in a manner to the inadequacy of the supplies, or which was not greatly aggravated by the insufficiency of means for preserving the health and hygienic efficiency of the soldier." And he adds, that at a time like the present, when an evident tendency exists to deny and make little of medical service to armies, it is opportune to solicit attention to the facts now related. Great results have been obtained as to the efficiency of soldiers and sailors through the observance of the rules of hygiene. Typhus fever has been extinguished, dysentery is seldom met with except in the tropics, smallpox is now a disease of rare occurrence, and scurvy at sea or on shore is seldom met with. Notwithstanding what Surgeon-General Gordon states, we are not sure that a tendency exists to deny and make little of medical service to armies. Both civilians, generally, and military officers are better informed in matters of sanitation and personal hygiene than in former years, and any practical recommendation brought forward by medical officers will, we believe, always receive attention. Such a state of matters as pictured by the late Lord Herbert and the late Sir Ranald Martin could scarcely now exist. Lord Herbert wrote: "When a medical officer goes to the general commanding, who, under a tropical sun, up a river surrounded by swamps, is feeding his troops with salt pork, and tells him that unless he gives them fresh meat and vegetables they will be down with fever and scurvy, he does no more than his duty. But if he is met by the man in authority by the rejoinder, 'Sir, when your advice is wanted it will be asked for,' the medical officer vows never again to expose himself to such a rebuke." And Sir Ranald Martin recites that when serving in one of the most pestilential countries of India, he suggested the most suitable arrangement for camping the men—the answer being, "I'll be d——d if I do!" Now here, says Sir Ranald, was no blundering lieutenant, but one of the most able field-officers I have ever known; yet such was his treatment of a grave matter of duty, the neglect of which, before the year was over, cost him his life. Times, however, are now happily changed. It is, however, for the medical officers and sanitarians to remember to be practical in their recommendations. It is useless making suggestions which cannot be carried out, such a course often resulting in ill-feeling between the military and medical departments, and in the idea, among the members of the later, that there is a tendency to ignore medical advice.

